# Neutrophils under the microscope: neutrophil dynamics in infection, inflammation, and cancer revealed using intravital imaging

**DOI:** 10.3389/fimmu.2024.1458035

**Published:** 2024-10-08

**Authors:** Andrew O. Yam, Arnolda Jakovija, Catherine Gatt, Tatyana Chtanova

**Affiliations:** ^1^ School of Biotechnology and Biomolecular Sciences, Faculty of Science, University of New South Wales, Sydney, NSW, Australia; ^2^ Immune Biotherapeutics Program, Garvan Institute of Medical Research, Sydney, NSW, Australia; ^3^ St Vincent’s School of Medicine, Faculty of Medicine, University of New South Wales, Sydney, NSW, Australia; ^4^ The Kinghorn Cancer Centre, St Vincent’s Hospital, Sydney, NSW, Australia

**Keywords:** neutrophil, intravital 2-photon microscopy, imaging, migration, infection, wound healing, cancer

## Abstract

Neutrophils rapidly respond to inflammation resulting from infection, injury, and cancer. Intravital microscopy (IVM) has significantly advanced our understanding of neutrophil behavior, enabling real-time visualization of their migration, interactions with pathogens, and coordination of immune responses. This review delves into the insights provided by IVM studies on neutrophil dynamics in various inflammatory contexts. We also examine the dual role of neutrophils in tumor microenvironments, where they can either facilitate or hinder cancer progression. Finally, we highlight how computational modeling techniques, especially agent-based modeling, complement experimental data by elucidating neutrophil kinetics at the level of individual cells as well as their collective behavior. Understanding the role of neutrophils in health and disease is essential for developing new strategies for combating infection, inflammation and cancer.

## Introduction

Neutrophils are the most abundant white cells in blood (1, 2) and are rapidly recruited from the bloodstream into inflamed tissues to combat pathogens and facilitate tissue repair (3–6). They wield an array of microbicidal molecules designed to destroy microorganisms through phagocytosis (7), release of neutrophil extracellular traps (NETs) (8), tissue remodeling and production of reactive oxygen species (ROS) (9–11).

Intravital microscopy (IVM) in combination with fluorescent neutrophil reporter mice (12–14) has enabled researchers to visualize neutrophils in real time (15, 16) and revolutionized our understanding of the cellular and molecular processes underpinning neutrophil activity in response to wounds, infections, and cancers (3, 17, 18). Recently, computational modeling has emerged as a valuable way of simulating neutrophil behavior in various environments to predict their collective responses. This integration of imaging and computational techniques provides a new framework for studying neutrophils not only as individual entities but for understanding their collective behavior and how it relates to their functions (19–21). This review highlights recent insights obtained through IVM studies of neutrophils and computational modeling of their movement, offering predictive capabilities that inform both basic research and clinical applications.

## Visualizing neutrophils with IVM

Several different imaging modalities have been employed for IVM and are reviewed in (22–24). Although both confocal and multiphoton microscopy are widely used for *in vivo* imaging, multiphoton microscopy can be advantageous for longitudinal imaging due to its confined volume of excitation (and reduced tissue photodamage and light scattering) (25, 26). Exciting advances in tissue clearing agents suitable for *in vivo* applications suggest the possibility of even greater depth of tissue penetration that would significantly expand IVM capabilities (27).

Both *ex vivo* and *in vivo* approaches can be used to label neutrophils for IVM but while *ex vivo* labeling offers more flexibility, it may lead to neutrophil activation during labeling. On the other hand, fluorescent reporter mice allow for long-term imaging of unmanipulated neutrophils, but fluorescent labels are not always restricted to neutrophils complicating subsequent analysis and interpretation (**Table 1**).

**Table 1:** Visualizing neutrophils with IVM – *ex vivo* labels and *in vivo* reporters.

Label	Specificity	Comments
*Ex vivo* antibody labelling		
anti-Ly6G (28)	Neutrophils	Specific *ex vivo* labeling but may lead to neutrophil activation
anti-Gr1 (29)	Neutrophils, macrophages	*Ex vivo* labeling that is not restricted to neutrophils and may lead to neutrophil activation
*In vivo* reporter mice		
Ly6G “Catchup” (14)	Neutrophils	Transgenic reporter mice allow for longitudinal imaging of neutrophils over long periods of time. However, some reporter mice used to visualize neutrophils are not completely specific to neutrophils.
Myeloid related protein 8 (MRP8) (30)	Neutrophils, macrophages	
Lysozome M (LyzM) (12, 13, 31)	Neutrophils, macrophages, monocytes, dendritic cells	
Optical highlighters - photoconvertible (e. g., Kaede (32, 33) and Kikume (34)) and photoactivatable (e. g., PA-GFP (35)) transgenic mice	Not specific to neutrophils, further crosses required to mark neutrophils	Photoconvertible and photoactivatable reporters can be used to track neutrophil fate and migration between organs (18). These transgenic lines need to be adapted for neutrophil-specific labeling.
**Nanoparticles**	Neutrophils	• E.g., fluorescent magnetic cubes and clusters nanoparticles (36): detectable using magnetic resonance imaging (MRI) and taken up by neutrophils *in vivo* (can potentially be used for drug delivery and diagnostics). • CD11b labelled nanorods for labeling activated neutrophils (37).
**Radioactive isotope labeling**	Neutrophils	Nano-radiotracers such as ^68^Ga and a peptide ligand of FPR1, cinnamoyl-F-(D)L-F-(D)L-F (cFLFLF) (38) can be detected by positron emission tomography (PET) and MRI; allow for detection of neutrophils in site of inflammation.

## Neutrophils in infection and injury

Neutrophils are pivotal to the body’s immune response to infection and injury (39, 40). Within minutes after injury, neutrophils infiltrate the site of inflammation in response to danger-associated molecular patterns (DAMPs) from damaged cells or pathogen-associated molecular patterns (PAMPs) from microorganisms (41, 42). There they release an array of anti-microbial agents as well as NETs containing DNA, histones, and proteases (43, 44). While neutrophils are best known for their essential roles in pathogen clearance, they also play an important role in wound healing after injury (45, 46).

### Neutrophil recruitment to the site of inflammation

Neutrophils circulate in a non-activated state until endothelial activation triggers their recruitment to sites of infection through a series of steps including tethering, rolling, adhesion, crawling, and transmigration (47). Their recruitment involves selectins on endothelial cells binding to ligands on neutrophils, such as P-selectin glycoprotein ligand-1 (PSGL-1), initiating tethering and rolling (48). This is followed by the engagement of integrins like lymphocyte function-associated antigen 1 (LFA-1) and Mac-1 with intercellular adhesion molecule-1 (ICAM-1) on endothelial cells, which strengthens adhesion and enables neutrophils to crawl and transmigrate into inflamed tissues (49).

Efficient navigation through capillary networks is crucial for optimal neutrophil recruitment to injury sites. To prevent congestion and ensure smooth passage through narrow vessels, neutrophils can alternate between branches at capillary bifurcation (50). This behavior is regulated by interactions between consecutive neutrophils, with the first neutrophil in one capillary branch altering the migration of the following neutrophil towards the other branch through a mechanism driven by chemoattractant gradient perturbation and hydraulic resistance (50). This alternating migration helps distribute neutrophil traffic uniformly at bifurcations of capillaries and ensures swift arrival at sites of damage without causing congestion in narrow vessels (50).

Subsequent trans-endothelial migration involves temporary spikes in calcium levels inside neutrophils as they squeeze through the vessel walls in live mice (51). These calcium spikes are triggered by a sensor protein called Piezo1. When it is activated, it leads to the production of hypoxic inducible factor 1α (HIF1α), which then turns on NADPH oxidase 4 (NOX4) and helps produce ROS to kill bacteria during infections (51).

Neutrophil recruitment into tissue is a crucial step in immune defense against infections, as evidenced by the findings that impaired extravasation through the basement membrane in neutrophils lacking mammalian sterile 20-like kinase 1 may contribute to the severe immune defect observed in patients with mammalian sterile 20-like kinase 1 (MST1) deficiency (52, 53).

The importance of regulating neutrophil migration in circulation was also underscored in recent studies of severe acute respiratory syndrome coronavirus 2 (SARS-CoV-2) infection. IVM revealed that neutrophils accumulated in the microcirculation of the lungs and brain of SARS-CoV-2 infected mice (54). This accumulation was associated with endothelial activation, local inflammation, and the formation of large platelet aggregates, particularly in the brain. These findings suggest that neutrophils contribute to the localized vascular and inflammatory changes observed in SARS-CoV-2 infection, highlighting their importance in the pathogenesis of coronavirus disease 2019 (COVID-19)-related complications (54).

### Neutrophil migration in inflamed tissues

Following transmigration neutrophils enter inflamed tissues and transition to directed migration towards the inflammatory foci called chemotaxis (55). For example, during focal necrosis in the liver, neutrophils were guided by a multistep process involving ATP release from necrotic cells, which activates the inflammasome, triggering neutrophil adhesion (56). Subsequently, their migration toward the injury site was directed by a chemokine gradient, but signals such as formyl peptides could override this gradient, ensuring that neutrophils are precisely guided into necrotic tissue (56). This finely tuned mechanism allows neutrophils to navigate through healthy tissue without causing unnecessary damage, focusing their action on damaged areas. Experiments in zebrafish and mouse neutrophils show that during chemotaxis neutrophils employ a ‘search and run’ strategy, where an initial actin-driven orientation is followed by rapid actin flows, enabling them to navigate tissue gradients effectively (57).

The orchestrated movement of neutrophils toward the attractant results in their focal accumulation and formation of dynamic clusters, termed ‘neutrophil swarms’ (3, 55). Since its discovery in 2008, neutrophil swarming has been observed in more than 30 inflammatory conditions, including sterile inflammation and infections by bacteria, parasites, viruses, and fungi (3, 55, 58, 59). In fact, swarming is a conserved function in tissue inflammation and is an integral part of wound healing.

The initial conceptual framework of swarming emerged from observations of dynamic cooperative behavior among neutrophils during intracellular parasitic *Toxoplasma gondii* (*T. gondii*) infection (3). Initially, “pioneer” neutrophils detected damage and then signaled to recruit additional neutrophils from distances greater than 70 μm, forming a swarm (3). These swarms were either transient or persistent, with the latter potentially leading to early granuloma formation and contributing to tissue remodeling by displacing collagen fibers (3).

Further insights into the molecular pathways governing neutrophil swarming have been gained through IVM studies of genetically modified and transgenic neutrophils in mice and zebrafish (57, 60–62). Leukotriene B_4_ (LTB₄) has emerged as key requirement for initiating neutrophil swarming (60). Upon detecting tissue or cell damage, neutrophils rapidly release LTB₄ in response to elevated intracellular calcium levels (63). This triggers a second wave of neutrophils, amplifying recruitment and forming clusters to isolate the injury or infection from healthy tissue (63). Neutrophil chemoattractant synthesis in response to tissue damage is triggered in response to “calcium alarm” signals propagated through direct contact among pioneer swarming neutrophils *via* connexins (proteins forming gap junctions in the membranes of adjacent cells) (61). Connexins such as Cx43 enhance the synthesis of chemoattractants, coordinate neutrophil swarming and promote wound sterilization in the damaged area (61). Another trigger for swarm initiation is the release of cellular components (including chromatin, gasdermin, neutrophil elastase and myeloperoxidase) by pioneer neutrophils during NETosis (62). An earlier study differentiated between two types of NETosis: “suicidal” NETosis where neutrophils release NETs and undergo apoptosis, and “vital” where neutrophils remain motile and viable for some time after NETosis (64). However, it appears that, at least in this case, neutrophils undergoing “suicidal” rather than “vital” NETosis served as swarm initiators (62).

Regulation of the later stages of neutrophil swarming is still poorly understood but a recent study used a swarming-on-a-chip platform to demonstrate that inhibiting the Arp2/3 complex (which is crucial for regulating actin cytoskeleton dynamics) leads to cell death in densely clustered neutrophils within swarms (65). The pentose phosphate pathway plays an important role in supporting the viability and functionality of neutrophils during swarming by metabolically regulating the growth of neutrophil clusters and ROS production (65).

Although several studies have demonstrated how neutrophils can amplify swarming, there is considerably less clarity about how this self-amplifying behavior is turned off. In an elegant set of experiments, Lammermann and colleagues showed that neutrophils regulate their swarming behavior by desensitizing themselves to chemoattractants through a mechanism dependent on G protein-coupled receptor (GPCR) kinases (66). Specifically, neutrophils lacking GPCR kinase 2 (GRK2) were unable to desensitize to swarm-specific chemoattractants, although their response to other chemoattractants like LTB₄ and CXCL12 remained unaffected (66). Interestingly, GRK2-deficient neutrophils displayed increased mobility and greater infiltration into lymph nodes infected with *Pseudomonas aeruginosa (P. aeruginosa)*, yet this increased presence paradoxically correlated with a reduced ability to clear the infection, highlighting the complex regulation of neutrophil functions during swarming (66).

Other immune cells can also help to limit the extent of neutrophil swarming. For instance, in a microlesion laser injury model targeting the murine peritoneal serosa (a scenario that simulates single cell death), resident tissue macrophages extended their membrane processes to cloak the injury site, effectively preventing neutrophil swarming (67). In contrast, macrolesion laser injuries (which involve larger areas of damage), triggered neutrophil swarming because the resident macrophages were unable to cloak the injured site (67). This dichotomy in neutrophil response to different types of injury illustrates how neutrophil activity can be finely tuned to the specific context, further emphasizing the importance of balanced regulation to avoid excessive tissue damage.

In contrast to macrophages, degranulating mast cells (MCs) can exploit neutrophil swarming to perpetuate allergic inflammation (68). Upon releasing LTB₄, MCs attract swarming neutrophils and trap them within themselves forming structures called MC intracellular traps (MITs). This process represents a novel twist on neutrophil swarming, where instead of isolating damage or pathogens, neutrophils are retained by MCs, contributing to sustained inflammation in allergic tissues (68). Together these studies reveal that while neutrophil swarming is crucial for an effective immune response, dysregulation of this process can lead to excessive neutrophil activity without necessarily improving infection clearance, indicating the need for a delicate balance in neutrophil function to ensure optimal immune outcomes.

### Neutrophils in wound repair

Neutrophils play crucial roles in tissue repair beyond their immediate inflammatory response (39, 69, 70). They clear cellular debris, release growth factors like vascular endothelial growth factor A (VEGF-A) and matrix metalloproteases (e.g., MMP-8, MMP-9) to promote tissue regeneration and vascularization, and recruit macrophages for subsequent stages of repair (39, 71, 72). In a model of sterile liver injury, neutrophils contributed to revascularization and new collagen deposition by expressing the peptidase cathepsin C, which is essential for activating other neutrophil proteases (45).

Neutrophils also facilitate tissue repair by actively transporting extracellular matrix components from surrounding connective tissues to injured organs (73). This repair process is facilitated by the upregulation of collagen-binding integrins and the activation of heat shock factors, which enhanced the capacity of neutrophils to support tissue integrity during the healing phase (73). These studies highlight the critical role of neutrophils not only in initiating tissue repair but also in supporting long-term regeneration through mechanisms that facilitate both vascular and tissue remodeling.

In skin injury models nociceptive sensory neurons have recently emerged as a key regulator of neuro-immune interactions. During the healing process, these neurons communicate with immune cells in the skin through the neuropeptide calcitonin gene-related peptide (CGRP) (74). This neuropeptide regulates neutrophil and macrophage phenotype and turn-over, promoting tissue repair in skin and muscle injuries. Mechanistically, CGRP induces the expression of thrombospondin-1 (TSP-1) in these immune cells, limiting their accumulation and accelerating clearance in response to inflammatory cytokines. It also promotes neutrophil clearance via efferocytosis and shifts macrophages toward an anti-inflammatory, pro-repair phenotype (74).

While neutrophils are crucial for tissue repair, their activities can also lead to unintended tissue damage, particularly when their responses become dysregulated. For instance, during a methicillin-resistant *Staphylococcus aureus* (MRSA) infection, neutrophils released extracellular traps (NETs) in the liver vasculature, which, while trapping bacteria, also inflicted significant liver damage (75). Although DNase can remove the DNA component of NETs, it was less effective against harmful elements like histones and neutrophil elastase, which continued to damage tissue. Similarly, in a zebrafish tail fin regeneration model, neutrophil removal led to faster healing when CXCL8, a neutrophil recruiting chemokine, was knocked down, underscoring neutrophils’ dual role in both promoting repair and contributing to tissue injury (46). Understanding the factors that regulate their function is crucial for promoting wound healing in pathological and physiological conditions.

### Neutrophils in vascular disease

**Figure 1:**
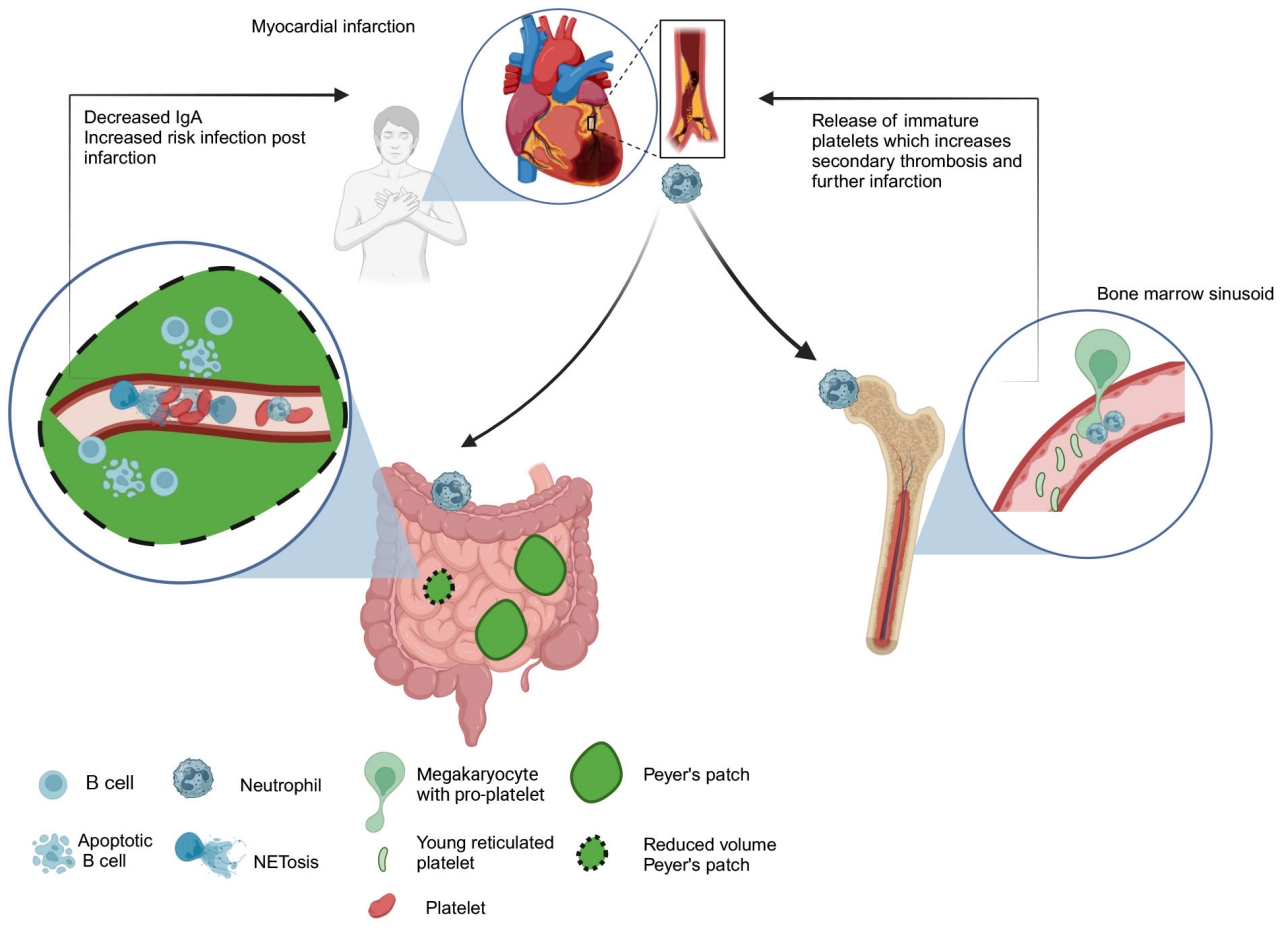
Neutrophil response to myocardial infarction. Systemic inflammation induced by stroke/myocardial infarction triggers NET release by circulating neutrophils. Increased circulating DNA levels lead to platelet aggregation in the vasculature causing microvascular thrombosis of Peyer’s patches. This could deprive them of nutrients and oxygen leading to the loss of IgA producing cells in Peyer’s patches and represents one potential mechanism contributing to increased risk of infection in patients post-infarction (76). Neutrophils may also increase the risk of secondary thrombosis after myocardial infarction by premature release of reticulated platelets from the bone marrow sinusoid. (77). Neutrophils regulate platelet production by “plucking” pro-platelets from megakaryocytes. This process becomes dysregulated following infarction and excessive release of prothrombogenic immature platelets leads to elevated risk of vascular thrombosis events post infarction (77).

Recent animal studies have linked neutrophils to systemic complications following vascular ischemia, such as strokes and myocardial infarctions (76, 77). One type of complication post-stroke is the increased risk of infections, and in clinical studies, stroke patients have lower serum IgA levels compared to healthy controls (78). Tuz et al., highlight an intriguing link between neutrophils and IgA levels. They noted a reduction in serum IgA in mice following stroke or myocardial infarction (76). This corresponded to reduced volume of Peyer’s patches in these animals (one of the sites of IgA production) as well as increased B cell apoptosis (**Figure 1**). The loss of IgA-producing plasma cells was linked to circulating DNA released during NETosis by neutrophils (which are activated by systemic inflammation post-stroke). Platelet aggregations induced by NETs may trigger oxygen and nutrient reduction and impact B cell survival in Peyer’s patches. The study suggests that neutrophil depletion or NET disruption could prevent IgA loss in patients and reduce the risk of infection.

In steady state neutrophils facilitate continuous release of platelets by “plucking” on megakaryocytes to drive platelet production but Petzold et al., showed that following myocardial infraction neutrophils can drive excessive release of prothrombogenic immature platelets, which increase risk of vascular thrombosis events post infarction (**Figure 1**) (77). Considering the critical roles that neutrophils play in mediating systemic effects following stroke and myocardial infarction, manipulating their number or activation state is emerging as a novel therapeutic strategy to reduce thromboischemic events.

### Neutrophil fate

Although most neutrophils are thought to undergo apoptosis at the site of inflammation (79), accumulating evidence suggests that tissue neutrophils can have several other fates. For instance, in zebrafish and mouse models, neutrophils can migrate back into the vascular system after completing their function in inflamed tissues in a process called “reverse neutrophil migration” (45, 80–82). In a sterile liver injury model, instead of being phagocytosed neutrophils exited the injured tissue and re-entered the bloodstream *via* reverse transendothelial migration (rTEM) and then entered the lungs before eventually returning to the bone marrow, where they underwent apoptosis (45).This dynamic process facilitated restoring tissue integrity in the liver by removing activated neutrophils (45). Similarly, in a zebrafish model of spinal cord injury resolution of inflammation was aided by neutrophil transmigration away from the site of injury to various tissues (83).

However, activated neutrophils reentering the vasculature can contribute to systemic inflammation (84). This was observed following ultraviolet B (UVB) light exposure, where fluorescently labeled neutrophils emigrated from the skin (84). A subpopulation of these neutrophils exhibited rTEM, localizing in the kidney and contributing to tissue inflammation and injury (84).

During infections, neutrophils can also use the lymphatic system to egress from inflamed skin and transport antigens to draining lymph nodes in a process dependent on CD11b and CXCR4 (18). This mechanism may facilitate cellular communication between the injury site and lymphoid organs, thereby modulating the adaptive immune response. Another function of neutrophils in lymph nodes is to intercept and neutralize pathogens. For example, in the study looking at *Staphylococcus aureus* (*S. aureus*) infection, lymph node neutrophils prevented microbial dissemination to peripheral organs (85). Taken together these studies provide a more nuanced understanding of how tissue neutrophils can perpetuate not only local but also systemic inflammation through reverse transmigration and lymphatic migration extending their capacity to regulate subsequent innate and adaptive responses beyond the initial site of inflammation.

## Neutrophils in tumors

Neutrophils infiltrate most solid tumors where they can either contribute to tumor growth and progression or stimulate anti-tumor immunity and tumor growth inhibition (86, 87). Recently, single cell RNA sequencing technology has significantly advanced our understanding of these seemingly contradictory roles by identifying different neutrophil subpopulations with distinct functional phenotypes (88–94). IVM can complement this approach by visualizing the dynamics and cellular interactions of the different intra-tumor neutrophil populations.

Analysis of the very early stages of oncogenesis in zebrafish revealed that neutrophils (as well as macrophages) formed cytoplasmic tethers and phagocytosed the transformed cells (6). As occurs in response to tissue injury, neutrophil recruitment to the tumors was dependent on hydrogen peroxide signaling (95). Inhibiting hydrogen peroxide reduced myeloid cell recruitment and the number of transformed cells suggesting that inflammatory signals from cancer cells recruit myeloid cells to support transformed cell development.

Even outside tumors, neutrophils can contribute to cancer metastasis by creating niches for circulating tumor cells. For instance, neutrophils activated by lipopolysaccharide (LPS) acted as a bridge/linker between circulating tumor cells and the hepatic sinusoid, contributing to metastases in the liver (96). Consistent with this observation, neutrophil depletion decreased the number of metastases (96, 97). Induction of NET formation in murine sepsis models revealed NETs as a potential mechanism whereby neutrophils trap circulating tumor cells in hepatic sinusoids (97, 98). Others have observed that metastatic murine 4T1 breast tumor cells induced NET formation in the lungs even in the absence of infection (99), suggesting that tumor cells can induce NETs thereby building their own metastatic niche. Conversely, NETs’ inhibition with neutrophil elastase inhibitor led to decreased hepatic and pulmonary metastases, while a similar effect was observed in mice deficient in a key enzyme required for NET formation (100), peptidyl-arginine deiminase type 4 (PAD4) (70).

IVM has provided additional clues about the influence of the tumor microenvironment (TME) on neutrophil dynamics and function (17, 101). In the murine oropharyngeal cancer (MOPC) model, intratumor neutrophils were found to move at lower velocity than peri-tumor neutrophils, suggesting that signals within the TME regulated their motility (101). As the tumors developed, neutrophils further decreased their velocity and directionality. Notably, peri-tumor but not tumor neutrophil recruitment was CXCR2-dependent suggesting that several distinct pathways govern neutrophil recruitment to the TME.

**Figure 2:**
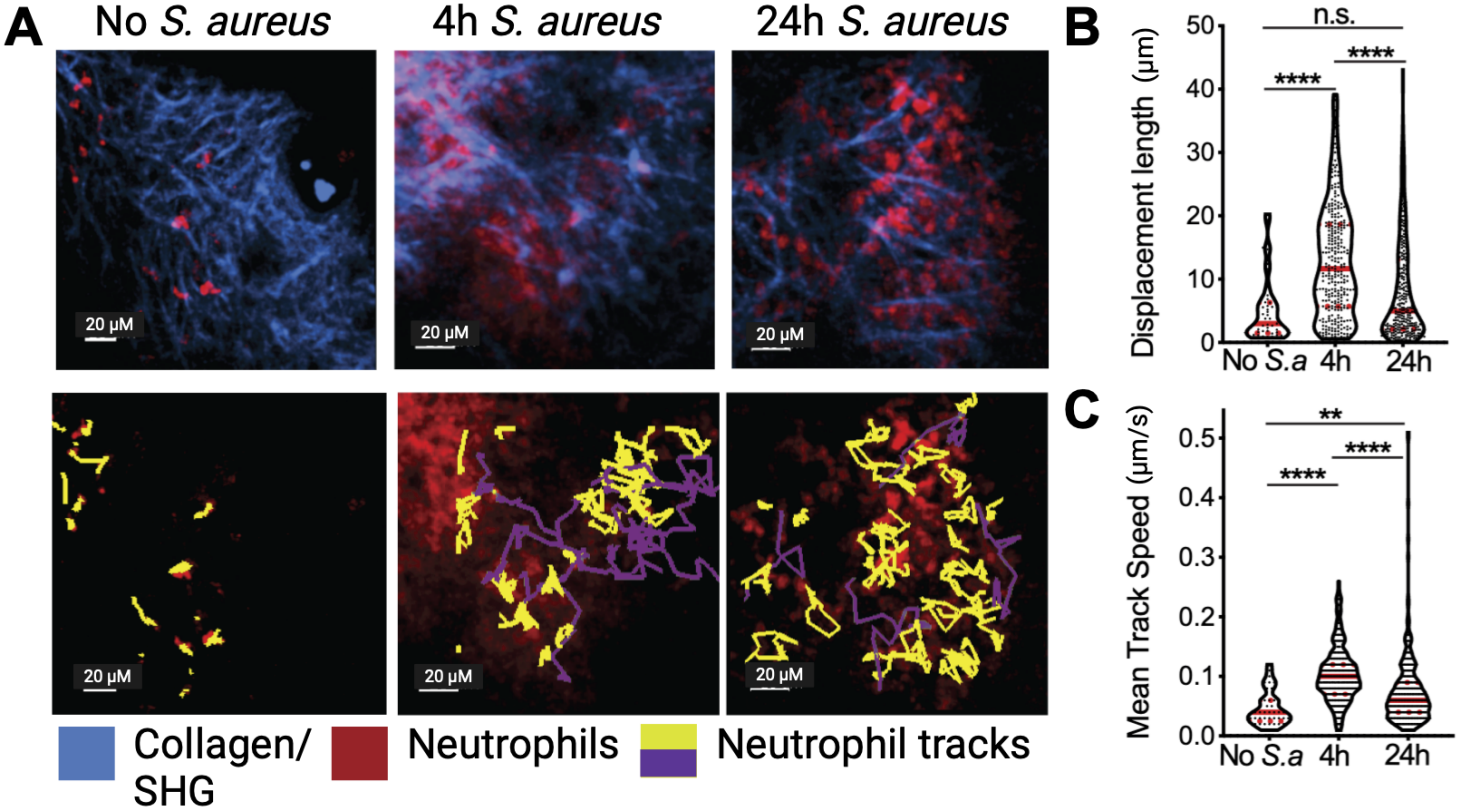
Tumor neutrophil dynamics *in vivo*. **(A)** Neutrophils (red) were visualized in a steady state or treated with intratumor injection of *S. aureus* bioparticles (*S.a*) for 4 and 24 h in Lewis Lung Cancer (LLC) tumors using intravital two-photon microscopy. Yellow tracks indicate neutrophils with confined motility, and purple tracks indicate migratory neutrophils. Second Harmonic Generation (SHG)/collagen - blue. Bar represents 20 µm. **(B)** Track displacement length of intratumoral neutrophils. **(C)** Mean track speed of intratumoral neutrophils. Data from at least 4 independent imaging experiments per time point were analyzed using a one-way ANOVA with Dunn’s correction for multiple comparisons **(B, C)**. Median and quartiles are shown. **P ≤ 0.01, ****P ≤ 0.0001; n.s. not significant. Adapted from Yam et al. (17).

Limited motility was also observed in neutrophils infiltrating Lewis lung carcinoma (LLC) tumors (17) (**Figures 2A–C**). However, tumor neutrophil number and velocity were dramatically increased when an infectious stimulus was introduced into the TME (**Figures 2A–C**). Within 4 hours of the introduction of *S. aureus* bioparticles into tumors, neutrophils increased their speed and displacement (**Figures 2B, C**), but in contrast to what was previously observed in infections, tumor neutrophils did not form dynamic swarms. By twenty-four hours after *S. aureus* bioparticle injection neutrophil motility was significantly reduced compared to the four-hour time point, suggesting that chronic inflammation in the TME limits their motility. In addition, neutrophils were observed to interact with tumor cells and tumor cells adjacent to neutrophil clusters formed blebs, suggesting that they were undergoing cell death.

Neutrophils in contact with tumor targets have been found to have a number of tumoricidal mechanisms that include releasing ROS (102–104) or neutrophil elastase (ELANE) (105, 106). They also prime CD8+ T cells with tumor associated antigens and can kill opsonized tumor antigens by antibody-dependent cellular cytotoxicity (ADCC) (107). IVM has shown that antibody-opsonized cancer cells can be removed by neutrophils via trogocytosis, a process in which immune cells acquire a membrane from another cell by an endocytic process (108, 109). Once neutrophils have trogocytosed tumor cells, the latter lose cellular integrity and die. Understanding how neutrophils destroy opsonized tumor cells may improve therapeutic antibody approaches for tumor treatment.

IVM has also contributed to understanding the mechanisms by which neutrophils can modulate cancer therapies. The use of anti-vascular endothelial growth factor (VEGF) was proposed as a possible new form of anti-tumor therapy (110). However, in zebrafish models, anti-VEGF therapy increased neutrophil infiltration, which remodeled tumor collagen to create a metastatic niche (111).

c-MET signaling has been used as an oncogenic target for patients with cancers. Inhibition of c-MET decreased neutrophil recruitment into tumors and their draining lymph nodes and improved T cell activation (112). An IVM-based approach showed that T cell activation was suppressed in the absence of c-MET inhibition by direct contact between T cells and neutrophils. Neutrophil mobility in the vascular space was also impaired in metastatic tumor mouse models (113). This was thought to be due to conformational inactivation of its β_2_-integrin (Mac-1/CD11b and LFA-1/CD11a), which was recapitulated by treating mice with granulocyte colony stimulating factor (GCSF). Based on these findings, it was proposed that the paraneoplastic effect of GCSF produced endogenously by tumors would cause vascular congestion impairing neutrophil vascular mobility, thereby blocking CD8+ T cell infiltration. In other words, neutrophils, by blocking effective CD8+ T cell infiltration of tumors, could promote resistance to checkpoint inhibitor therapies. In the cancer space, imaging technologies have broadened our understanding of how neutrophils interact with cancer cells and contribute to tumor progression and metastasis, underscoring their significance as potential targets for innovative cancer therapies.

## Modeling neutrophil behavior *in silico*

**Figure 3:**
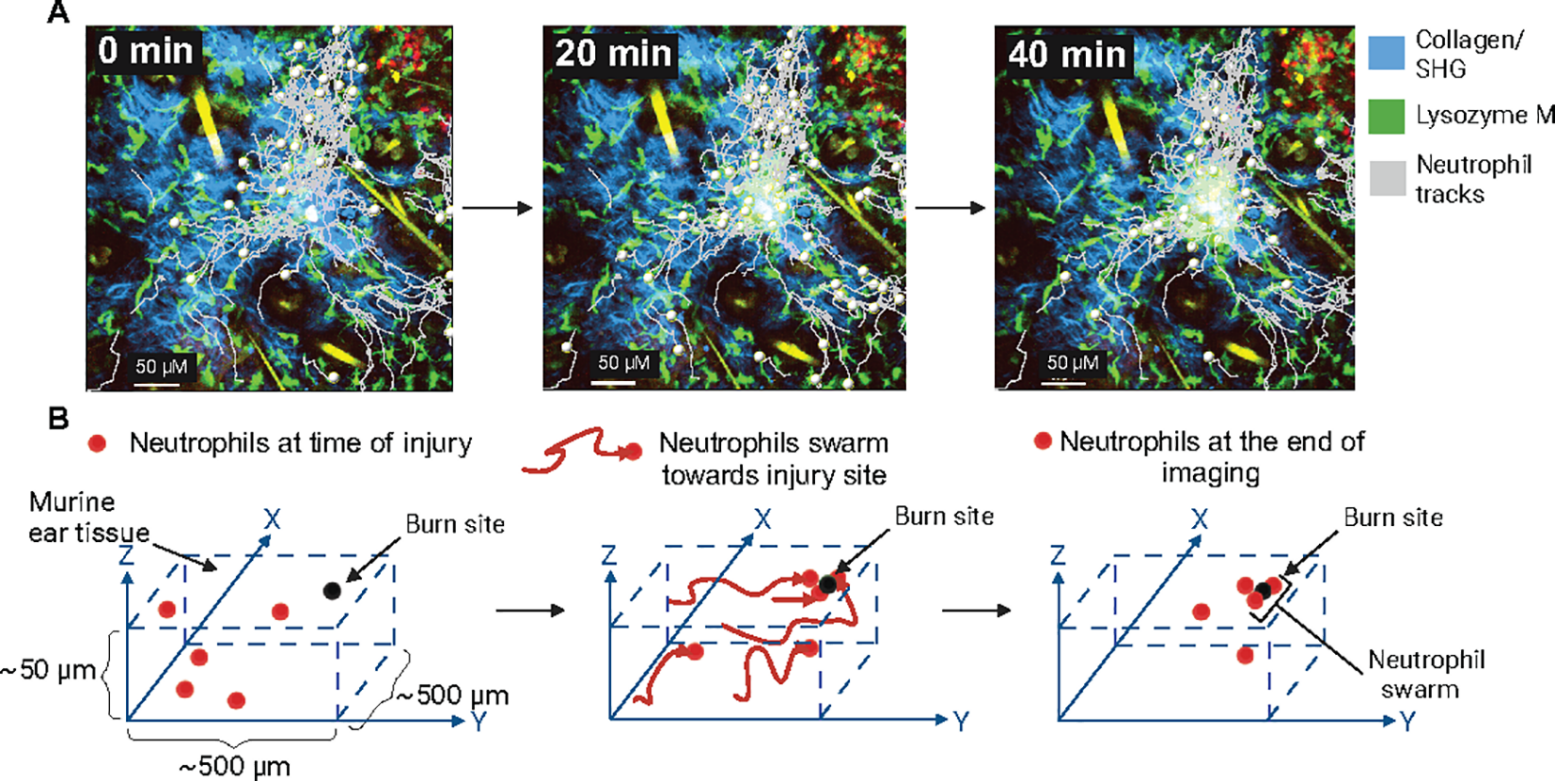
Neutrophil swarming and intravital imaging of neutrophil dynamics **(A)**. Lysozyme M (LysM) positive neutrophils (green) migrating towards the site of sterile laser murine ear injury (track marks grey) to form swarms. Second harmonic generation (blue), tick marks are 50 µM apart, and images were acquired by IVM. (unpublished data). **(B)** Schematic representation of neutrophil swarming towards a sterile laser injury. Neutrophils are tracked, moving towards the site of inflammation in 3D tissue.

Mathematical modeling and simulation techniques have significantly advanced our understanding of complex cellular behavior in immune defense (114). Statistical analysis and modeling of cell migratory patterns have been improved through the use of detailed, spatially resolved *in vivo* data that capture single cells moving through tissue and interacting with each other (115). IVM can record the dynamic movement of fluorescently labeled cells in their native environment (59) and the cell’s x, y, and potentially z coordinates, depending on the dimensions recorded, can be determined for each time point in the imaging sequence (**Figures 3A, B**). Using statistical analysis, we can then calculate various metrics of the cell’s movements, such as average turning speeds, velocities, and displacements or distances traveled (115, 116) (**Figures 2B, C**). This can be expanded to include detailed analysis of hundred cells (73, 77) from the same imaging volume to study how collective migration, such as swarming, is coordinated. This approach has shown that neutrophils can exhibit diverse behaviors in inflamed tissues, including a pathogenic sessile state localized near endothelial junctions, which contrasts with their typical migratory role (20). Notably, this study also demonstrated that neutrophil morphology, the center of mass, and the height-to-length ratio were linked to their behavior. Therefore, IVM coupled with statistical analysis can provide additional parameters to classify neutrophils and their associated function and help identify pathogenic and non-pathogenic states.

Following the quantitative analysis of IVM data, cell motility can create mathematical representations or ‘models’ to simulate and predict the movement of immune cell populations and their responses to environmental stimuli (117). One commonly used modeling approach to studying immune cell behavior is agent-based models (ABMs) (114). ABMs characterize discrete agents (for example, cells) with their own set of rules or behaviors (for example, velocity) and simulate the interactions of these agents within a defined environment. IVM data have been combined with the ABM approach to help understand leukocyte behavior, migration patterns, and search strategies in various contexts. Read et al. (21) found that the migration of neutrophils under inflammatory conditions was best captured by ABMs that account for differences in speed and direction changes within the population. They found that neutrophil populations are statistically heterogeneous in rotational and translational speeds, and directional persistence. Neutrophils had inversely correlated translational and turn speeds, meaning cells moving quickly do not perform large reorientations and vice versa. This pattern may stem from limitations in cytoskeletal remodeling or reflect the need to navigate environmental obstacles, necessitating slower movement to maneuver effectively around them. Similar findings were observed in the exploration patterns of T cells in larval zebrafish, which showed a broad distribution of speeds within cell populations and that, like neutrophils, fast-moving T cells make shallow turns while slow-moving T cells make bigger turns (118). These observations made from modeling T cell migration suggest that there is an actin-dependent intrinsic component of cells that jointly controls speed and directional persistence. Given the growing use of ABMs and IVM to model leukocyte migration behavior, there is significant potential to apply these models to study neutrophil dynamics in tumors, infections, and injuries.

Currently, IVM data often requires time-consuming manual analysis, which limits the number of parameters that can be analyzed (i.e., type and number of cells tracked). Application of models such as ABM has the potential to increase the throughput of data acquired through IVM. Others have trained computer learning networks with manually labeled images to track neutrophils (19). Refinement of neutrophil modeling and application of machine learning methodologies can provide tools for higher throughput spatial and kinetic subtyping of neutrophils.

## Technological challenges

However, developing reliable methods for statistical analysis and modeling immune cells using *in vivo* imaging data presents several challenges. The choice of quantitative metrics can influence which model best describes a cell’s motility (21, 119). Furthermore, *in situ* fluorescence microscopy, which is necessary to observe single cells over various spatial scales, faces technical limitations, including the need for micron-scale resolution over millimeter-scale fields of view (118). Long-term imaging can also cause photodamage to cells (120), altering their behavior and, therefore, negatively impacting the model’s validity. These constraints underscore the need for improved methods and complementary metrics in studying cell migration. For example, data from IVM modeling could be used to understand how to create organoid environments that better mimic *in vivo* physiological and pathological conditions (121). Combining single cell RNA-sequencing approaches with intravital imaging could help correlate *in vivo* heterogeneity with the diversity of neutrophil populations identified by single-cell sequencing. Although it is technically challenging to link an individual cell’s trajectory and gene expression, *in vivo* photolabeling could be used to mark cells that are swarming or exhibit specific motility patterns as a prelude to sequencing them.

## Discussion

Advances in IVM and fluorescent neutrophil-reporter animal models have provided a highly detailed window into the role of neutrophils in health and disease, which means there is a rich vein of neutrophil spatial-temporal data in response to different stimuli. IVM showed how neutrophils’ functions (therefore, their temporal and spatial kinetics) are shaped by their tissue environment. In some conditions their spatial kinetics are highly orchestrated and conserved, as evidenced by their ability to form swarms in a staged fashion. In other pathophysiological conditions, such as neutrophil responses to cancers, we are yet to identify a signature balletic movement. Detailed insights into neutrophil motility patterns and interactions could enable the design of targeted therapies to modulate their activity, potentially improving outcomes in inflammatory and infectious conditions as well as solid tumors.
